# Evidence of gene-nutrient interaction association with waist circumference, cross-sectional analysis

**DOI:** 10.1186/s12889-024-19127-z

**Published:** 2024-07-10

**Authors:** Anwar H. AlBaloul, Jennifer Griffin, Alexandra Kopytek, Paul Elliott, Gary Frost

**Affiliations:** 1 Department of Community Medicine and Behavioural Sciences, Faculty of Medicine, Kuwait University, Safat, Kuwait; 2 Section of Nutrition, Faculty of Medicine, Imperial College London, London, UK; 3 Department of Epidemiology and Biostatistics, School of Public Health, Imperial College London, London, UK

**Keywords:** Diet score, Waist circumference, Genetic risk score, Obesity, Gene-diet interaction

## Abstract

**Background and aims:**

Waist circumference (WC) is a significant indicator of body adiposity and is associated with increased mortality and morbidity of cardiovascular diseases. Although, single nutrient intake and candidate genes were previously associated with WC. Little is known about WC association with overall diet quality, genetic risk score and gene-nutrient interaction. This study aims to investigate the influence of overall diet quality and multiple WC-associated single nucleotide polymorphisms on WC. In addition to investigating gene-nutrient interaction association with WC.

**Methods:**

This study explored cross-sectional data from two large sample-size studies, to provide reproducible results. As a representation of the UK population, the Airwave Health Monitoring Study (*n* = 6,502) and the UK-Biobank Cohort Study (*n* = 171,129) were explored for factors associated with WC. Diet quality was evaluated based on the Mellen Index for Dietary Approaches to Stop Hypertension (Mellen-DASH). The genetic risk score for WC (GRS-Waist) was calculated by screening the population genotype for WC-associated single nucleotide polymorphisms. Multivariate linear regression models were built to explore WC association with diet quality and genetic risk score. Gene-nutrient interaction was explored by introducing the interaction term (GRS-Waist X Mellen-DASH score) to multivariate linear regression analysis.

**Results:**

The prevalence of high WC (Female > 80 cm, Male > 94 cm) was 46.5% and 51.7% in both populations. Diet quality and genetic risk score of WC were significantly associated with WC. There was no evidence of interaction between GRS-Waist, DASH diet scores and nutrient intake on WC.

**Conclusion:**

This study’s findings provided reproducible results on waist circumference association with diet and genetics and tested the possibility of gene-nutrient interaction. These reproducible results are successful in building the foundation for using diet and genetics for early identification of those at risk of having high WC and WC-associated diseases. In addition, evidence on gene-diet interactions on WC is limited and lacks replication, therefore our findings may guide future research in investigating this interaction and investigating its application in precision nutrition.

**Supplementary Information:**

The online version contains supplementary material available at 10.1186/s12889-024-19127-z.

## Introduction

Globally there is a noticeable increase in the prevalence of obesity and obesity-related diseases [1]. This increase in obesity is in parallel with the increase in the prevalence of diabetes [2] and cardiovascular diseases [3]. The UK National Health Service estimated over £5 billion per year is spent on obesity-related health treatment [4]. Waist circumference (WC) is recognised as a surrogate measure for obesity and an indicator of cardiometabolic risk [5–7]. Although WC is significantly associated with diabetes and dyslipidaemia, little research has addressed the multiple risk factors associated with WC, including genetics, diet, and the interaction between the two [3, 8].

Recent genetic studies examined the effect of WC-associated genes on WC. To date, 92 loci are associated with WC [9]. The biological mechanism of all 92 loci is still under investigation. However, studies explained the biological role of a few loci on WC. For example, polymorphisms within the MC4R gene were positively associated with WC among the European population [10]. This WC-MC4R relationship was found to be regulated by the MC4R gene association with total energy and dietary fat intake [11]. Polymorphisms within ZNRF3 and VEGFA genes were associated with adipocyte lipolysis affecting waistline measurement [12]. In general, studies explored the association between a single genetic variant with WC, overlooking the aggregated effects of multiple genetic markers.

According to the World Health Organisation, overall diet quality is key in managing and preventing obesity [13]. The Dietary Approach to Stop Hypertension (DASH) is a diet quality index characterised by high consumption of vegetables, fruits, and dietary fibre, and low intake of red meat and saturated fatty acids [14, 15]. Several studies have demonstrated the significant associations between DASH diet components and WC. For example, fruit and vegetable intake was negatively associated with WC [16]. On the other hand, energy intake from fat was positively associated with WC [17]. Although single components of the DASH diet were associated with WC, a few studies have addressed WC association with the overall DASH diet score, within the UK population.

The literature provides strong evidence for genetic and diet associations with WC. To date, little is known about the aggregated effect of multiple genetic markers and overall diet quality association with WC, in addition to the interaction between the two. Therefore, our primary aim is to investigate the associations of genetic WC predisposition and overall diet quality exposure with WC. Our secondary aim is to investigate genetic and diet interaction association with WC. Motivated to explore the role of diet and nutrients in modifying the association between genetic variants and WC. Finally, the lack of robust and reproducible results is the main challenge facing gene-diet interaction studies. Therefore, we will replicate our analysis across two UK-based studies.

## Methods

### Study population

Our cross-sectional study was nested within the Airwave Health Monitoring Study (AHMS). The AHMS is a longitudinal cohort study to investigate the health of 42,112 police force participants in the United Kingdom [18]. Data were collected from 2007 to 2012 and details for recruitment and screening are described elsewhere [18]. Our nested study included 6,502 AHMS healthy participants. Participants answered a general questionnaire on lifestyle and medical history, in addition to completing a 7-day dietary record. Participants also provided blood samples for genotyping. Trained nurses collected Anthropometric data such as weight, height, and WC. We included participants with available genetic and dietary data. Our exclusion criteria were based on the following, first, we excluded pregnant participants due to the effect of pregnancy on overall body weight and WC. Second, we excluded participants of non-white British ethnicity. This is because the selected WC-associated genes were significant among British-white descent and were not explored across other ethnicities. A flow chart illustrating the study’s final sample size is shown in supplementary FIGURE S1.

We replicated our analysis across the UK-Biobank Cohort Study. The UK-Biobank Cohort Study (UK-Biobank) is a large cohort study that determined the distribution of diseases and disease risk factors within the UK population. It collected data from 500,000 adults in the UK [19]. Data included: anthropometrical measurement, blood biochemistry, genetics, and diet. Anthropometrical measurements were collected by trained personnel in the UK-Biobank centres. Dietary data were collected through a repeated 24-hour dietary recall. Our UK-Biobank cross-sectional sample included 171,129 participants. We followed a similar inclusion and exclusion criteria as the AHMS. A flow chart illustrating the study’s final sample size is shown in supplementary FIGURE S2. We chose the UK-Biobank cohort to replicate our analysis because we believed that a larger sample size may improve the precision and generalisation of our results [20].

### Dietary data collection and diet quality assessment

We collected dietary data from a validated self-administered 7-day food diary [21]. Participants were given instructions on recording food intake, including cooking methods and weight estimation [22]. We used Dietplan version 6 software for dietary data entry and nutrient analysis. Quality control checks and data cleaning processes were followed to ensure the quality of dietary data entry [22]. The dietary data output sheet included nutrient intake per quantity of food items over the recorded days. Nutrients were divided over the number of recorded days to estimate the average daily intake.

We assessed diet quality using the Mellen Dietary Approaches to Stop Hypertension index (Mellen-DASH) score [15]. The Mellen-DASH score evaluates the intake of 9 nutrients (total fat, saturated fat, protein, fibre, cholesterol, calcium, magnesium, potassium, and sodium). It combines these into one score reflecting the overall diet quality [15, 23]. Each nutrient is scored: 0 points for not reaching the targeted amount, 0.5 for intermediate and 1 for meeting recommended consumption. Thus, the summary score varies between 0 and 9. The Mellen-DASH score components were calculated using the average 7-day intake per participant. A higher score indicates high adherence to the Mellen-DASH diet recommendation and better diet quality.

Sodium and total cholesterol intake were missing from UK Biobank dietary data. The following steps addressed the missing nutrients; first, dietary sodium intake was calculated by using the UK-Biobank 24-hour urinary sodium output variable. This was achieved using a Brown et al. validated equation to estimate dietary sodium intake in micrograms from urinary sodium output [24]. Second, the score was rescaled to assess the intake of 8 nutrients instead of 9 to account for missing total cholesterol intake.

### Single nucleotide polymorphisms selection and genetic risk score

The Genetic Investigation of Anthropometric Traits (GIANT) consortium identified 219 single nucleotide polymorphisms (SNP) related to WC in European-descent populations [25]. Since Genome-Wide Association Studies (GWAS) scans the whole genome for potential WC-associated SNPs, we selected SNPs that reached a significant genome level (*P*-value < 5 × 10 − 8) [26]. This has led to the exclusion of 27 non-significant SNPs. SNPs with a minor allele frequency (MAF) above 0.1 and SNPs were selected [26].

The LDlink version 4.0.1 was used to identify Linkage disequilibrium (LD) between selected SNPs [27]. The LDlink was set to the following criteria: distance 500 kb, population panel European ancestry, and LD threshold r2 = < 0.01 [28]. The minimum length distance was defined as 500 kb, to ensure the non-random association between alleles within the same loci. This has led to the exclusion of 100 SNPs, with linkage equilibrium and MAF < 0.1. After excluding SNPs with non-significant genome levels and LD pruning, the final analysis included 92 SNPs. SNPs’ genetic information is shown in Supplementary Table S1.

For this study, we obtained previously genotyped data. Participants’ samples were genotyped using the Illumina HumanCore Exome-12v1-1 BeadChip array. Genotyped data is available upon request.

A genetic risk score estimates the cumulative number of the risk alleles of a specific trait presented within an individual’s genotype. Our genetic risk score for WC (GRS-Waist) was calculated from 92 WC-associated SNPs. At each SNP, the participant’s genotypes were coded based on the number of risk alleles into 0, 1 and 2, then weighted by multiplying the number of risk alleles by each estimated coefficient [29]. Our calculated GRS-Waist is an indication of the cumulative number of the risk alleles associated with WC presented in each participant. A high GRS-Waist indicated a higher number of WC risk alleles leading to a higher genetic predisposition of having a high WC.

### Covariates

Our primary outcome was WC, collected by trained nurses according to the WHO protocol [30]. We also collected height and weight to measure participants’ body mass index (BMI). BMI and WC were categorised according to the WHO anthropometric classification [30]. BMI was classified into three categories (Normal = 18–25 kg/m2, Overweight = 25–30 kg/m2, Obese = > 30 kg/m2). Waist circumference was classified into two categories across gender, high WC (Female: = > 80 cm, Male = > 94 cm), and very high (Female = > 88 cm, Male = > 102 cm).

We considered the effect of other confounding variables, such as Physical Activity Level (PAL) and smoking. PAL was assessed using the short version of the International Physical Activity Questionnaire (IPAQ) [31]. Physical activity was calculated and categorised by the metabolic equivalent of the task in minutes per week (MET minutes a week); high (> 60 min/d of at least moderate-intensity activity), medium (> 30 min/d of at least moderate‐intensity activity) and low (no activity or less than medium category) [31]. The Smoking variable was categorised into smokers and non-smokers, where previous and current smokers were combined.

### Statistical analysis

We used R statistical software version 3.6.1 for all analyses; the statistical significance threshold was set at *P* < 0.05. Continuous variables were tested for normality by Shapiro–Wilk test and were normally distributed.

We compared both cohorts (AHMS and UK-Biobank) first by two proportion Z-test to determine the difference in sociodemographic and the prevalence of WC and BMI classifications. Second, an unpaired t-test was to investigate the difference in dietary intake and GRS-waist.

### WC association with nutrient intake, diet quality and GRS-waist

We used multivariable linear regression models to explore WC association diet quality score and nutrient intake. The Mellen-DASH score and nutrient intake were primary predictors. Nutrients (percentage energy from Fat, Protein, and carbohydrates) and fibre were selected based on significant associations observed in previous studies [16, 32]. Association with fruit and vegetable intake was also investigated due to significant observation in previous studies [17]. The adjusted quality model included age, sex, BMI, PAL and total energy intake (kcal/day). Models were adjusted for BMI and energy intake because it was previously shown that this adjustment may implicate an association independent of whole-body adiposity and a dietary association with other dietary attributes than energy intake [33, 34].

We used multivariable logistic regression models to explore WC association GRS-waist. Our primary outcome was high WC classified according to the WHO (high WC; Female > 80–88 cm, Male > 94 to 102 cm, very high WC; Female > 88 cm, Male > 102 cm). The GRS-waist group were the primary predictor, we stratified the study sample by GRS-waist median into a low GRS-Waist group below the median, and a high GRS-Waist group above the median. The adjusted GRS-Waist model included age, sex, BMI and PAL.

### GRS-waist - Diet score interactions

We examined evidence of gene-diet interaction (GRS-waist X Nutrients or DASH score tertiles). Dietary variables were categorised into tertiles because previous interaction studies observed that adopting a high-quality dietary pattern offset GRS-BMI association with BMI [35]. Individual interaction models were built for each nutrient and DASH score. Adjusted interaction models included age and sex, BMI, PAL, and energy intake (kcal/day). The likelihood ratio test was used to test for evidence of interaction between the full interaction model and the reduced model.

## Results

### Sample characteristics

Descriptive statistics of the 6,502 AHMS participants are shown in Table 1. The sample included 60.6% male participants, with mean population age of 40.8 years. The prevalence of high WC and very high WC were 27.2% and 19.3%, respectively. The prevalence of overweight and obese BMI was 47.5% and 19.3%, respectively. The AHMS descriptive dietary analysis is shown in Table 2. The average Mellen-DASH score was 2.5 ± 1.4, and the average portions of fruits + vegetable intake were 2.8 ± 1.5.

**Table 1:** Characteristics of participants in the Airwave Health Monitoring Study and the UK-Biobank Study

	AHMS (*n* = 6,502)			UK-Biobank (*n* = 171,129)	
Characteristic		**Percent**	**N**	**Percent**	**N**
Sex					
Male		60.6%	3,942	44.9%*	94,315
Female		39.4%	2,560	55.1%*	76,814
WHO anthropometric classification ^a^					
BMI					
Normal (≥ 18–25 kg/m^2^)		33.2%	2,156	38.3%*	65,588
Overweight (≥ 25–30 kg/m^2^		47.5%	3,090	42.0%*	33,747
Obese (≥ 30 kg/m^2^)		19.3%	1,256	19.7%*	71,794
Waist circumference ^a^					
High WC (female ≥ 80 cm – male ≥ 94 cm)		27.2%	1,770	25.8%*	44,126
Very high (female ≥ 88 cm – male ≥ 102 cm)		19.3%	1,255	25.9%*	44,326
Physical activity level ^b^					
Low		15.4%	1,004	26.1%*	44,601
Moderate		32.5%	2,113	48.2%*	82,468
High		52.1%	3,385	25.7%*	44,060
Smoking status					
Non-smoker		90.9%	5,910	57.1%*	97,648
Smoker		9.1%	590	42.7%*	73,143

^a^ World Health Organisation anthropometric classification

^b^ International Physical activity questionnaire

* Statistically significant *p*-value < 0.05, two proportion Z-test

**Table 2:** Descriptive dietary statistics across the Airwave Health Monitoring Study and UK-Biobank

	AHMS (*n* = 6,502)		UK-Biobank (*n* = 171,129)	
Nutrient	**Mean**	**SD**	**Mean**	**SD**
Mellen-DASH score ^a^	2.5	1.4	3.4 *	1.3
GRS-Waist	94.1	6.0	93.9 *	6.0
Fat % ^b^	26.2	8.2	39.2 *	8.0
Protein % ^b^	17.1	3.3	18.9 *	4.2
Carbohydrates % ^b^	47.2	6.9	57.7 *	9.9
Fibre g/1000Kcal	9.3	2.7	9.5 *	3.4
Fruits and vegetable ^c^	2.8	1.5	7.8 *	4.3

^a^ Mellen index for Dietary approach to stop hypertension [15]

^b^ Macronutrient intake is presented as the average percentage of energy from macronutrients

^c^ food groups as exposure variables expressed as the number of portions categorised based on the DASH diet 100 g/ one portion

* Statistically significant *p*-value < 0.05, t. test comparing two independent variables

Descriptive statistics on 171,129 UK-Biobank participants are shown in Table 1. The sample included 44.9% male participants, with mean population age of 56.2 years. The prevalence of high WC and very high WC were 25.8% and 25.9%, respectively. The prevalence of overweight and obese BMI were 42.0% and 19.2%, respectively. The UK-Biobank descriptive dietary analysis is shown in Table 2. The average Mellen-DASH score was 3.4 ± 1.3, and the average portions of fruits + vegetable intake were 7.8 ± 4.3.

We observed a significant difference between both cohorts. First, UK-Biobank included a significantly high proportion of Females than AHMS. Second, AHMS included a higher proportion of high WC than UK-Biobank, whereas UK-Biobank included a higher proportion of normal BMI. Third, AHMS included a higher proportion of participants engaging in high PAL, and a lower proportion of participants engaging in moderate PAL, in comparison with UK-Biobank. Also, we observed a significant difference across the mean of all nutrients and the Mellen-DASH score. In addition to a significant difference in the mean GRS-Waist.

### WC status association with a genetic predisposition to WC and diet quality

Our findings showed that the Mellen-DASH score is negatively associated with WC (Table 3). The estimated coefficient in WC; AHMS: was − 0.22 (95% CI -0.31 to -0.13) per 1 increase in the Mellen-DASH score (*P* < 0.0001), and UK-Biobank − 0.23 ( 95% CI -0.22 to -0.18).

**Table 3:** waist circumference associations with dietary exposure in the Airwave Health Monitoring study and the UK-Biobank study^a^

	AHMS		UK Biobank	
	β	95% CI	β	95% CI
Mellen-DASH score ^b^	-0.22 *	-0.31, -0.13	-0.23 *	-0.22, -0.18
Fat % ^c^	0.01 *	-0.007, 0.03	0.017 *	0.01, 0.017
Protein % ^c^	-0.06 *	-0.09, -0.02	-0.02 *	-0.03, -0.02
Carbohydrates % ^c^	-0.03 *	-0.05, -0.02	-0.02 *	-0.026, -0.021
Fibre g/1000 Kcal	-0.10 *	-0.15, -0.06	− 0.06 *	-0.07, -0.06
Fruits and vegetable ^d^	-0.25 *	-0.33, -0.17	-0.04 *	-0.05, -0.04

* *P* < 0.001

^a^ Adjusted model: sex, age, BMI, PAL, and total energy intake per day

^b^ Mellen index for Dietary approach to stop hypertension [15]

^c^ Macronutrient intake is presented as the average percentage of energy from macronutrients

^d^ food groups as exposure variables expressed as the number of portions categorised based on the DASH diet 100 g/ one portion

After adjustment for age, sex, BMI and PAL, the percentage of energy intake from protein and carbohydrates, fibre intake (g/1000 kcal) as well as fruit and vegetable intake (portions/day), were negatively associated with WC. These associations were also explored across UK-Biobank data as shown in Table 3. Replicated analyses showed that percentage energy intake from protein and carbohydrates, fibre intake as well as fruit and vegetable intake, were negatively associated with WC.

**Fig. 1:**
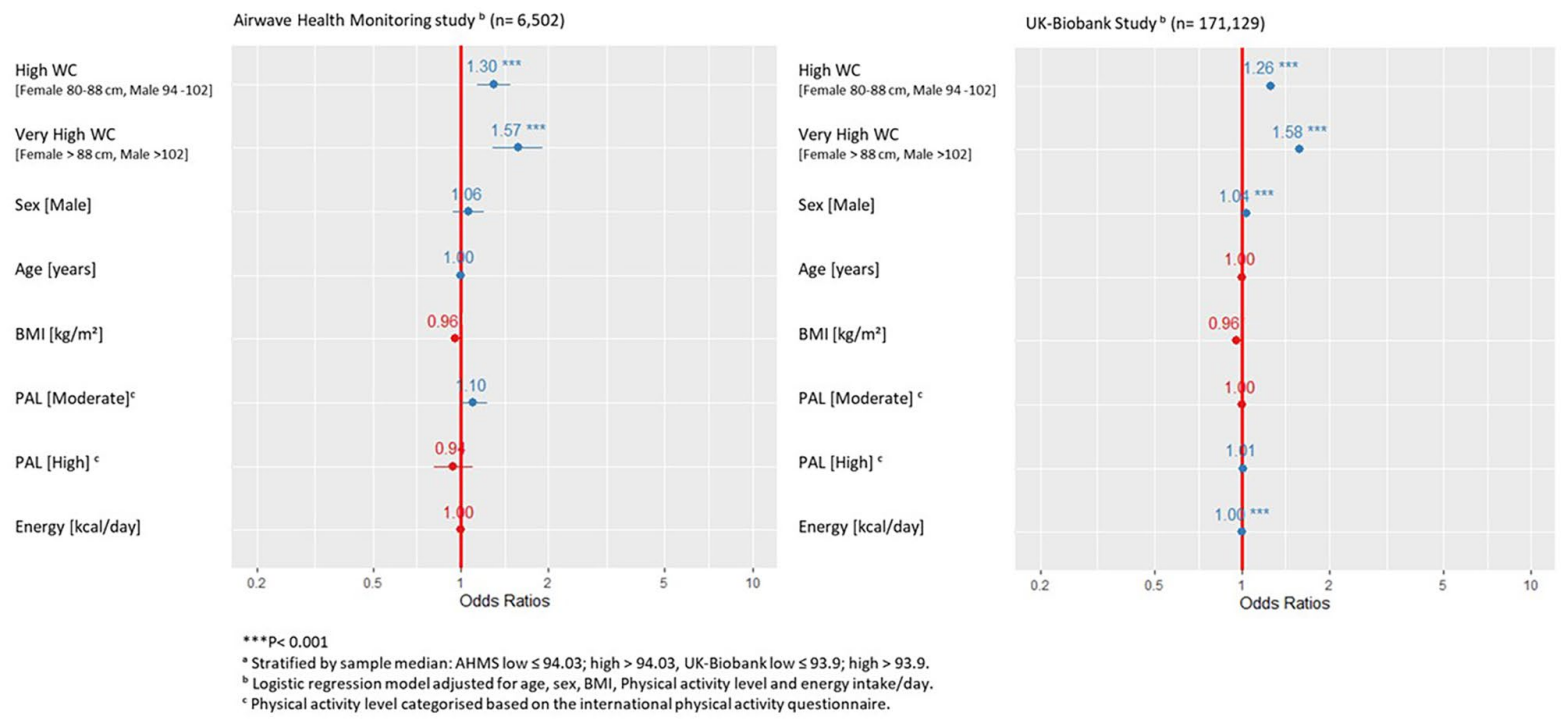
Multiple logistic regression analysis for factor associated with waist circumference genetic risk score groups [high and low GRS-waist]^a^

Our AHMS findings showed that the high GRS-waist group (GRS-Waist > 94.03) had1.30 (95% CI,1.13 to 1.47) times greater risk of high WC, and 1.57 (95% CI, 1.28 to 1.91) times greater risk of very high WC, than the low GRS-waist group (Fig. 1). Also, our UK-Biobank findings showed that high GRS-waist group (GRS-Waist > 93.93) had 1.26 (95% CI, 1.2 to 1.29) times greater risk of high WC, and 1.58 (95% CI, 1.52 to1.63) times greater risk of very high WC, than the low GRS-waist group (Fig. 1).

### GRS waist X Diet score/Nutrients interaction

Genetic and nutrient interaction effect on waist circumference, across diet score and macronutrient tertiles, is shown in Table 4 for AHMS and Table 5 for UK-Biobank populations. At first, we observed a significant interaction between GRS-waist and carbohydrate intake within the AHMS data, and between GRS-waist and fibre intake within the UK-Biobank data. However, a likelihood ratio test showed no evidence of interaction between GRS-waist, and those nutrients on waist circumference.

**Table 4:** The GRS-waist ^a^ and diet interaction effect on waist circumference across diet score or nutrient ^b^, Airwave Health Monitoring Study (*n* = 6,502) ^c^

Mellen DASH score	Q1	Q2	Q3	LST *P*-value ^d e^
	REF	0.006 (0.04,0.05)	0.01(-0.03,0.06)	0.51
Percentage energy from fat	Q1	Q2	Q3	
	REF	-0.01(-0.06,0.03)	-0.02	0.73
Percentage energy from protein	Q1	Q2	Q3	
	REF	0.015 (-0.03,0.06)	-0.005(-0.05,0.04)	0.65
Portions of fruits and vegetable	Q1	Q2	Q3	
	REF	-0.01(-0.06,0.03)	-0.003(-0.05,0.04)	0.86
Percentage energy from carbohydrates	Q1	Q2	Q3	
	REF	-0.04*(-0.09, − 0.001)	-0.02(-0.07,0.02)	0.13
Dietary fibre 100 g/100 kcal	Q1	Q2	Q3	
	REF	0.005 (-0.04 to 0.053)	-0.009 (-0.05 to 0.03)	0.59

*P < 0.05

^a^ genetic risk score indicator for the number of risk alleles associated with waist circumference

^b^ Mellen-DASH score and nutrient intake stratified into tertiles

^c^ β coefficient (95% confidence intervals) for interaction results (GRS-waist X dietary tertiles), adjusted for age, sex BMI and physical activity

^d^ Likelihood ratio test comparing two models with and without the interaction. Reduced model without interaction variable, adjusted for GRS, diet score or nutrient, age, sex, BMI and physical activity

^e^ Likelihood ratio test *P*-value

**Table 5:** The GRS-Waist ^a^ and diet interaction effect on waist circumference across diet score or nutrient ^b^, UK-Biobank (*n* = 171,129) ^c^

Mellen DASH score	Q1	Q2	Q3	LRS *P*-value ^d e^
	REF	-0.006 (-0.02,0.01)	-0.008(-0.03,0.01)	0.69
Percentage energy from fat	Q1	Q2	Q3	
	REF	0.01(-0.01,0.03)	0.005(-0.01,0.02)	0.59
Percentage energy from protein	Q1	Q2	Q3	
	REF	-0.008 (-0.03,0.01)	0.006 (-0.01,0.02)	0.39
portions from Fruits and vegetable	Q1	Q2	Q3	
	REF	0.01(-0.009,0.03)	0.004 (-0.017,0.02)	0.53
Percentage energy from carbohydrates	Q1	Q2	Q3	
	REF	0.01(-0.005, 0.03)	-0.0001(-0.02, 0.02)	0.23
Dietary fibre (grams/1000 kcal)	Q1	Q2	Q3	
	REF	0.02*(0.0008, 0.04)	0.008(-0.01,0.03)	0.12

*P < 0.05

^a^ genetic risk score indicator for the number of risk alleles associated with waist circumference

^b^ Mellen-DASH score and nutrient intake stratified into tertiles.

^c^ β coefficient (95% confidence intervals) for interaction results (GRS-waist X dietary tertiles), adjusted for age, sex BMI and physical activity

^d^ Likelihood ratio test comparing two models with and without the interaction. Reduced model without interaction variable, adjusted for GRS, diet score or nutrient, age, sex, BMI and physical activity

^e^ Likelihood ratio test *P*-value

## Discussion

In two samples of nearly 177,631 UK adults, we found that genetic predisposition to WC and dietary exposure were independently associated with WC. We observed that those with a high GRS-waist are more likely to have a high WC in comparison with a low GRS-waist group. We found no interaction between dietary intake and genetic predisposition to WC.

Worldwide changes in dietary patterns over the past decades are associated with the rapid rise in the prevalence of obesity [36]. Several pieces of evidence proposed improving adherence to healthy dietary patterns to reduce the prevalence of obesity and obesity-associated outcomes [37, 38]. It was previously suggested that diet quality scores are indicators for overall dietary intake, including nutrients and foods, which represent a wider input on dietary intake [39]. Additionally, studies showed that WC is a significant indicator of overall obesity, abdominal obesity and cardiometabolic risk [3, 5, 8]. The DASH diet favours the intake of food and nutrients that are protective against overall obesity and high WC, such as vegetables, fruits, legumes, and whole grains [17, 40]. Previous UK-based research showed a significant association between WC and components of the DASH diet index [16, 17]. However, limited data on the overall DASH diet score association with WC is available. Therefore, our findings proposed that DASH dietary patterns may be protective against high WC. Consistent with our findings, Romaguera et al. [16] one-year follow-up study showed that fruit and vegetable intake is significantly associated with lower WC. Koh-Banerjee et al. [17] also provided evidence supporting the negative association between dietary fibre intake and WC. Our findings and previous data may lead to a valuable interpretation that adopting DASH dietary recommendations may prevent WC gain and reduce its effect on developing cardiometabolic diseases.

The literature did not propose a precise mechanism explaining the association between DASH diet components and obesity. However, several factors could contribute to this association. First, The DASH diet is rich in fibre-containing food items, such as fruits, vegetables, and whole grains. It has been demonstrated that fibre is associated with increasing satiety, which might reduce fat and total caloric intake, leading to a lower risk of weight gain [41]. Second, the DASH diet favours a lower fat and saturated fat intake. Digested fatty acids are stored within adipose tissues for reserved energy. Excessive dietary fat intake leads to excessive fatty acids and triacylglycerols storage within adipocytes, leading to enlarged adipocytes and obesity [42].

To our knowledge, this study is the first study to investigate the distribution of WC genetic risk alleles and their association with WC in the UK population. Our study findings proposed that individuals with high GRS-Waist are 1.26 to 1.30 times more likely to be characterised with high WC (Females > 80 cm and Males > 94 cm). Also, our analysis proposed that individuals with high GRS-Waist are 1.57 to 1.58 times more likely to be characterised with a very-high WC (Females > 88 cm and Males > 102 cm). This evidence suggests the likelihood of having high WC is positively associated with the number of WC risk alleles presented within the individual genotype. Studies using genetic risk scores similar to the one used in our research have found a significant association between GRS-BMI and obesity. A previous UK-Biobank study showed a 1.41 kg/m^2^ increase in BMI for an increase in the number of BMI-associated risk alleles [43]. Another study showed that the GRS of BMI accounts for 2.2% of the variance in BMI [44]. Whereas the literature is limited in exploring the association between GRS-waist and WC. Our findings are consistent with GRS-BMI’s previous reports that a greater GRS of anthropometrical measurements is associated with a greater risk of having high WC or BMI. Therefore, our findings and prior data suggest that genetic variation might be responsible for regulating the difference in obesity measurements.

The mechanisms by which WC-associated SNPs are associated with a greater risk of having high WC are not fully explained yet. However, three SNPs included in our genetic risk score for WC (GRS-waist) have been linked to factors associated with obesity. First polymorphisms in the *MC4R* gene were associated with regulating energy and satiety [45]. Also, the number of risk alleles within the *MC4R* genotype was associated with high total fat and calorie intake [11]. Ollmann et al. suggested that the effects of *MC4R* polymorphisms on neuronal signalling in the hypothalamus are associated with increased hunger and higher caloric intake, which might lead to obesity [46]. Second, *ZNRF3* and *VEGFA* genes were recognised for their role in stimulating adipocyte lipolysis [12]. Polymorphisms within those genes were found to affect adipocyte signalling which reduces lipolysis, leading to increase fat storage and obesity [12]. Therefore, the aggregation of these markers by the GRS could explain the positive association between GRS-waist and WC observed in our study.

Comparisons between AHMS and UK-Biobank populations showed a significant difference in proportions of sexes, the prevalence of high WC and overweight/obese BMI, and physical activity levels. Serval factors could explain this difference. First, AHMS included more males than females; on the other hand, the UK-biobank study included more female than male participants. Due to differences in hormonal expressions, previous studies showed a significant difference in adiposity levels across sexes. Females are more likely to store fat with higher hip measurements than males and tendency to have a higher waistline [47, 48]. Therefore, the significant difference in the proportions of sexes might lead to differences in the prevalence of anthropometric classification. Second, both cohorts observed a significant difference in physical activity levels. Occupational requirements could explain this difference; the AHMS recruited participants from police forces that might have affected physical activity. Finally, a significant difference was observed in dietary intake and diet quality. The difference in dietary assessment could explain this; AHMS assessed dietary intake through a weight-estimated 7-day food diary, and UK-biobank used multiple 24-hours recalls, which might lead to differences in estimating dietary intake [49]. Additionally, variation in nutrient intake might be observed across ages and sexes [50].

To our knowledge, this is the first investigation of the interaction between dietary intake and genetic predisposition of WC. We were motivated to explore this interaction on the basis that genetic obesity risk would be offset or modified by diet quality and nutrient intake [51]. We expected that the negative association between the Mellen-DASH score and WC would influence the relationship between genetic risk and WC. We found no evidence of this interaction. Although at first, the multivariable regression model showed evidence for this interaction, however, likelihood ratio test showed a non-significate effect. This could be explained by sample size power. Our findings are consistent with previous studies in which diet and genetic interaction did not affect anthropometrical measurements. Burgoine et al. explored the effect of GRS-BMI and fast-food outlet interaction on BMI [52]. After stratifying by BMI-category they concluded that exposure to fast-food outlets and high GRS-BMI was not associated with higher BMI [51]. After stratifying by sex another cross-sectional analysis observed no interaction between GRS-BMI and sugary beverage intake among male participants [53]. Their findings suggested that high exposure to sugary beverages and high GRS-BMI is not associated with having a high BMI. This evidence and our findings are consistence in reporting that gene-diet interaction is not associated with anthropometrical measurements. No evidence of interaction could be explained by proposing that genetic risk is not equally exposed to diet quality. i.e., individuals with low genetic risk might be exposed to a low diet affecting overall obesity or WC.

Alternatively, Qi et al. observed a significant interaction between sugar-sweetened beverages with the GRS-BMI among female participants [53]. Their results suggested that sugar-sweetened beverage intake is associated with the genetic predisposition to elevated BMI. Wang et al. explored the interaction between GRS-BMI and three dietary indices [51]. Their analysis showed that after 20 years of follow-up, adopting a high-quality dietary pattern offset GRS-BMI association with BMI [51]. The same follow-up analysis showed that for those with high GRS-BMI, one standard deviation increase in DASH scores has led to a significant reduction in BMI [51]. Qi et al. was the first paper to propose a possible mechanism behind the significant effect of gene-diet interaction on BMI [54]. They suggested that the possible underlying nutrigenomic mechanism is the effect of *FTO* and *MC4R* genetic markers on regulating appetite [54]. Both genetic markers affect appetite, which affects food consumption, leading to high energy intake and possible risk of obesity.

Diet and genetic interaction studies aimed to test similar hypotheses, however, they led to two different conclusions. Some studies reported significant evidence of genetics and diet interaction, and other reports were compatible with our study in findings of no evidence of this interaction [35, 52, 53]. Several factors could have led to this variation, such as differences in the statistical analysis used to determine this interaction and differences in dietary assessment methods. First, Qi et al. observed a significant interaction among females and stratifying by sex could account for variation in gene expression across sexes [53]. It was not possible to explore the interaction across sexes within the AHMS population, because that has led to reducing the sample size. Luan et al. suggested that observing a gene-environment interaction is highly affected by sample size [55]. Second, Qi et al. investigated the genetic interaction with single food items such as sugary beverages [53]. This dietary assessment is different from the method used to assess dietary intake in the AHMS and UK-Biobank. Finally, Wang et al. observed a significant GRS and DASH interaction across longitudinal data [35]. Therefore, the difference in study design might have led to variations in study outcomes. Also, this might propose that dietary effect on genetic markers might occur over time.

Furthermore, several studies observed significant gene-environment interactions among those with homozygous obesity risk alleles [56, 57]. Since GRS is an indicator for the number of risk alleles presented with a genotype, it is possible to indicate that a significant interaction might be observed across those with high GRS. Therefore, Wang et al. reported significant gene-diet interaction among those with the highest BMI-GRS quartile in comparison with lower BMI-GRS [51]. These reports may suggest that a significant interaction might be observed if genetic data are stratified by GRS. Therefore, inconsistency in dietary assessment and statistical models could explain the different outcomes observed in our study and other reports.

### Research implication

Our findings could be implemented in several research areas. First is the requirement for further research testing the feasibility of using GRS-waist to identify those at risk of high WC and WC-associated diseases early. Second, although we found no evidence of interaction, our results and prior studies highlight the necessity for further research to test interaction models.

### Strength and limitations

The main strength of our study is using standardised methods to assess dietary intake and diet quality in a large sample size. Multiple 24 h dietary intake and 7-day weighted dietary records were correlated with low random errors and bias and high precision [58]. We computed the GRS-wait out of 92 WC-associated SNPs, which considered the polygenic nature of WC. Trained nurses and researchers collected anthropometrical data (weight, height and WC) according to the WHO protocol [59].

Although our study had several strengths it was prone to limitations. Its cross-sectional design was limited in providing evidence of causality. It is possible that genetic factors may drive food behavioural selection. Anthropometrical-associated genetic markers were shown to interact with environmental factors such as (Physical activity, sleep duration, and time spent watching tv) [60], which suggests the importance of including these variables. Our statistical models were adjusted for physical activity; however, data on sleep duration and TV watching were not analysed. Diet has a prolonged effect on adiposity, whereas a snapshot of dietary intake may be limited to assessing dietary modification’s effect on WC risk alleles. Therefore, exploring follow-up dietary analysis may be more informative in assessing this interaction. Finally, our study BMI categories were not aligned with the WHO norms, which might affect direct comparability with other studies.

## Conclusion

Our study confirmed previous findings that diet quality is associated with obesity, and it is the first to determine WC association with the DASH diet across the UK population. Also, it is the first to determine the genetic predisposition of WC and gene-diet interaction association with WC. These findings are important drivers for further investigation of risk factors associated with the obesity epidemic and precision nutrition. In addition, these factors should be considered in public health promotions and health policy decisions. Although we found no evidence of interaction, we recommend further testing on interaction models and exploring this interaction across follow-up dietary data.

### Electronic supplementary material

Below is the link to the electronic supplementary material.

Supplementary Material 1

## Data Availability

All data are available throughout the Dementia Platform https://portal.dementiasplatform.uk/. Access available upon request from https://portal.dementiasplatform.uk/Apply. The genotyped datasets generated and analysed during the current study are available from the ICHTB Tissue Management Committee. Restrictions apply to the availability of these data, which were used under licence for this study. Data are available [ https://police-health.org.uk/applying-access-resource ] with the permission of ICHTB Tissue Management Committee.

## Abbreviations

AHMS: Airwave Health Monitoring Study
BMI: Body Mass Index
DASH: Dietary Approach to Stop Hypertension
GRS: Genetic Risk Score
GRS-Waist: Genetic Risk Score of Waist Circumference
WC: Waist Circumference
