# Supplementary material for: Evidence of gene-nutrient interaction association with waist circumference, cross-sectional analysis
Source: BMC Public Health. 2024 Jul 10;24:1842. doi: 10.1186/s12889-024-19127-z (PMC11234640; doi:10.1186/s12889-024-19127-z)
Supplement: Supplementary file 1 — Supplementary Material 1 [file 12889_2024_19127_MOESM1_ESM.pdf]

Evidence of gene-nutrient interaction association with waist circumference, cross-sectional analysis.

Supplementary Materials

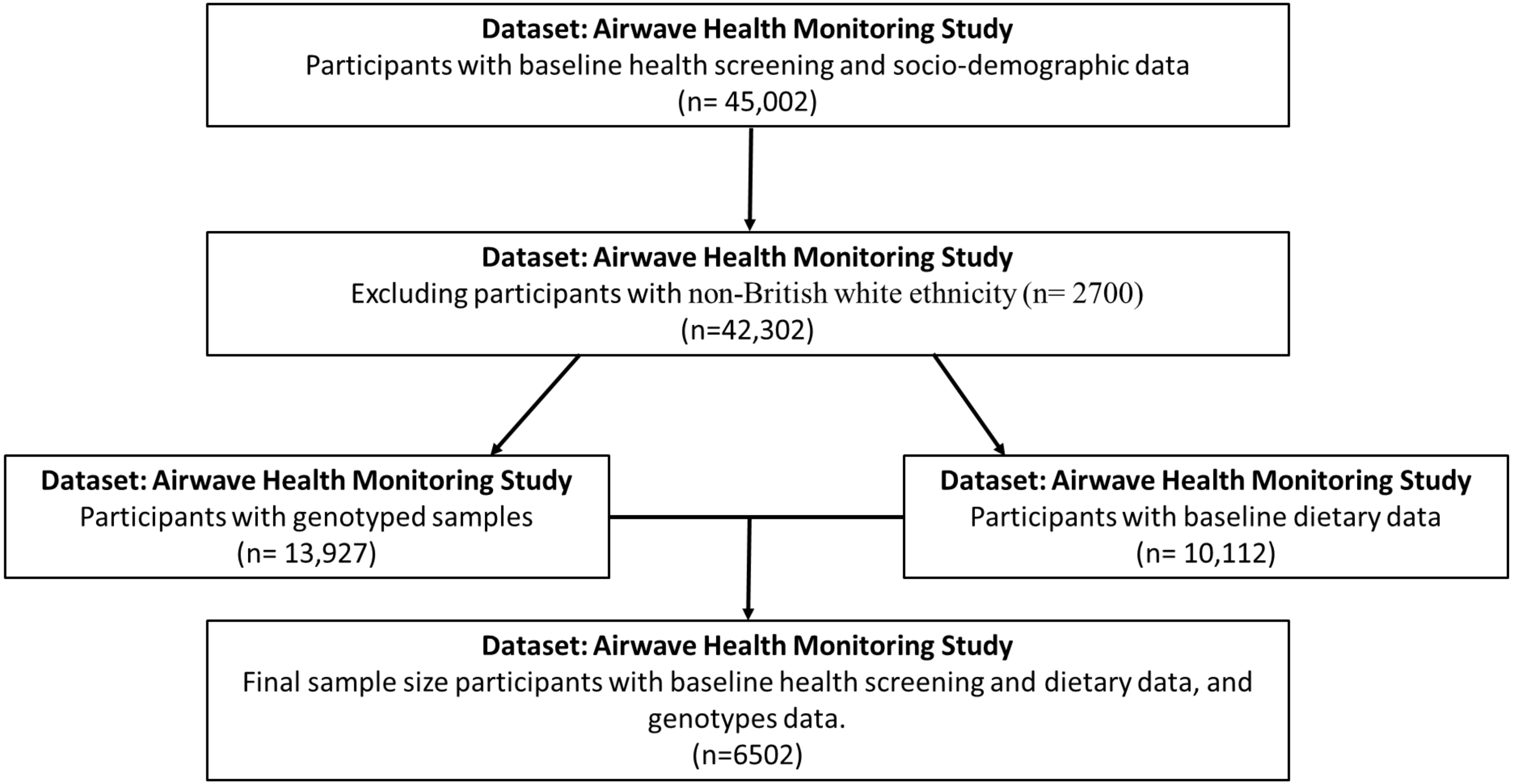

FIGURE S1 Flowchart illustrating inclusion and exclusion of AHMS participants and samples collected.

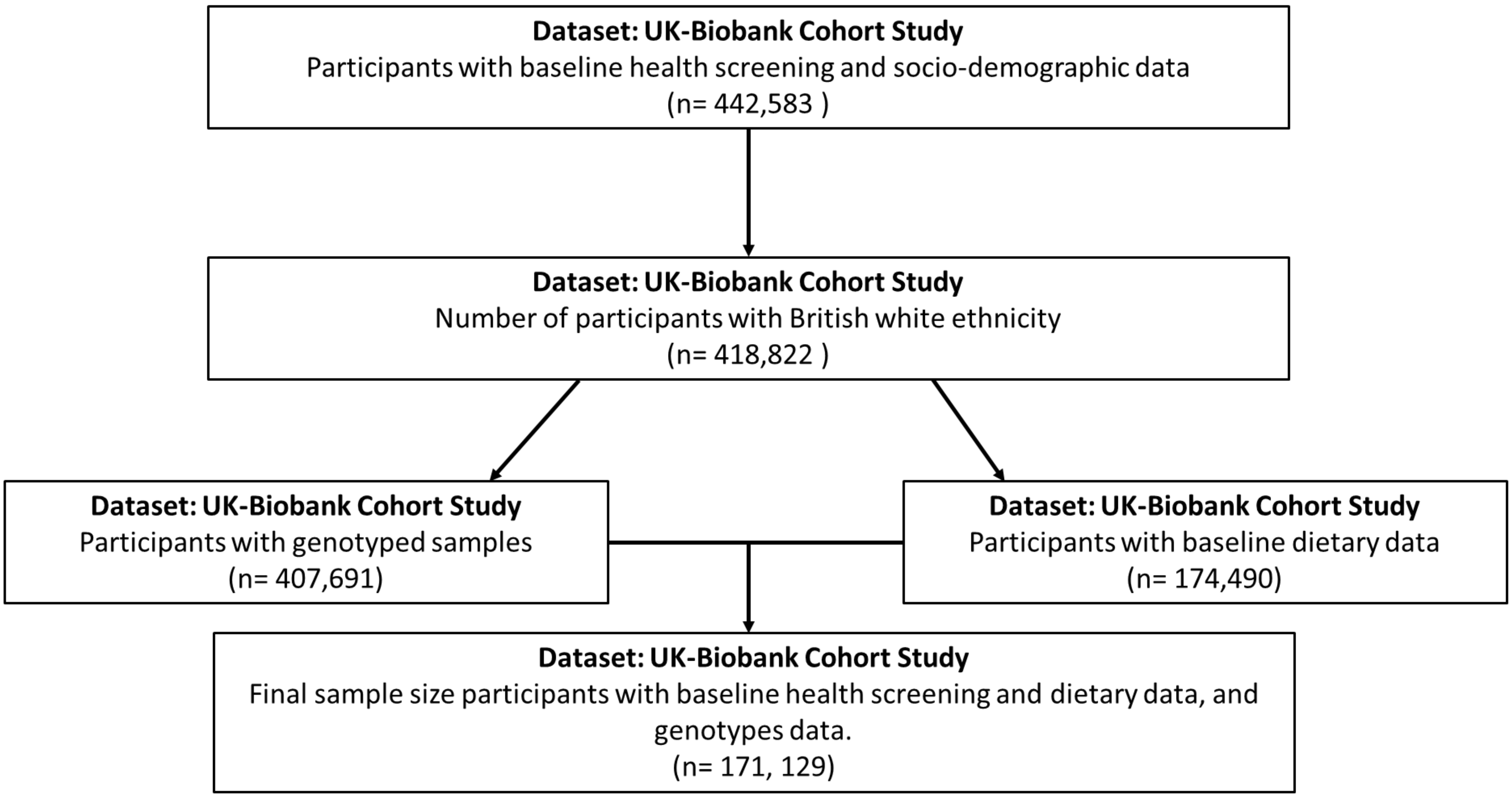

FIGURE S2 Flowchart illustrating inclusion and exclusion of UK-Biobank participants and samples collected.

Table S1 SNPs associated with waist circumference, reported by Genetic investigation for anthropometrical traits[1]

SNP rs number	Chromosome	Gene	Effective allele	Non-effective allele	Position built 37	β coefficient	P-value
**rs10041657**	5	FER	A	G	108152428	0.0251	2.88E-10
**rs10150088**	14	TRIP11	T	C	92503576	0.0201	6.82E-09
**rs10269774**	7	CDK6	A	G	92253972	0.0229	2.90E-08
**rs10516107**	5	CPEB4	A	G	173348156	0.0232	8.29E-11
**rs10771410**	12	PTHLH	T	G	28307204	0.0265	6.50E-13
**rs11144688**	9	PCSK5	A	G	78542286	0.0336	1.87E-08
**rs11205277**	1	SF3B4	G	A	149892872	0.0311	2.80E-14
**rs11695471**	2	DNMT3A	T	A	25457708	0.0299	1.60E-11
**rs12127195**	1	HLX	A	G	221309417	0.0211	7.67E-09
**rs12207675**	6	FILIP1	C	T	76237741	0.0333	7.10E-10
**rs12317176**	12	DNAH10	T	C	124404718	0.0211	3.69E-09
**rs12493901**	3	FNDC3B	G	A	171922055	0.0239	1.40E-11
**rs12608504**	19	JUND	A	G	18389135	0.0208	2.86E-08
**rs12679556**	8	MSC	G	T	72514228	0.0221	1.10E-08
**rs13210323**	6	ANKS1A	A	C	35005084	0.0216	1.45E-08
**rs1330**	11	NUCB2	T	C	17316029	0.0216	4.36E-08
**rs1482852**	3	LEKR1	A	G	156798294	0.0293	3.45E-14
**rs17396340**	1	KIF1B	A	G	10286176	0.0283	3.00E-08

**rs1776897**	6	HMGA1	G	T	34195011	0.0536	6.80E-17
**rs1812175**	4	HHIP	A	G	145574844	0.033	3.95E-13
**rs1879529**	15	ACAN	T	G	89414295	0.0239	2.86E-10
**rs1894633**	1	DNM3	G	A	172331059	0.0203	1.30E-08
**rs2030839**	15	ADAMTSL3	C	T	84583959	0.0381	2.81E-24
**rs2047937**	16	ZNF423	T	C	49864791	0.0186	4.67E-08
**rs2050157**	6	GPR126	G	A	142658162	0.0263	5.00E-12
**rs2052670**	2	KRT18P33	A	G	66218481	0.02	1.53E-08
**rs2071449**	12	HOXC5	A	C	54428011	0.0292	4.18E-14
**rs2124969**	2	ITGB6	T	C	160989486	0.0199	7.06E-09
**rs2214442**	7	ITGB8	A	G	20392787	0.0265	3.89E-09
**rs2294239**	22	ZNRF3	A	G	29449477	0.0191	3.49E-08
**rs2300149**	3	ITIH1	C	T	52822921	0.0206	6.60E-09
**rs2425170**	20	PHF20	G	A	34442672	0.0302	6.55E-09
**rs2580821**	2	DIS3L2	C	A	232804155	0.0433	2.00E-11
**rs2637030**	5	NDUFS4, ARL15	G	A	52976825	0.0221	1.10E-08
**rs272869**	5	SLC22A4	A	G	131677997	0.0212	6.68E-10
**rs2745353**	6	RSPO3	T	C	127452935	0.0293	7.88E-19
**rs3786897**	19	PEPD	A	G	33893008	0.0199	8.77E-09
**rs3791675**	2	EFEMP1	C	T	56111309	0.0358	3.21E-16
**rs3811964**	5	NPR3	T	G	32770099	0.0238	3.40E-10

**rs3814333**	1	GLT25D2	T	C	184007119	0.0287	2.40E-14
**rs3843467**	5	MAP3K1	T	G	55856375	0.0289	1.45E-09
**rs3862030**	10	SUFU	A	G	104327584	0.0206	5.83E-10
**rs4141278**	7	NFE2L3	T	C	25857525	0.0335	3.39E-15
**rs4246302**	15	ADAMTS17	A	G	100687967	0.0216	5.73E-09
**rs4308051**	18	CABLES1	G	T	20735461	0.0423	7.87E-23
**rs4378999**	3	DOCK3	T	A	51208646	0.0358	4.10E-08
**rs4542783**	19	MYO1F	T	C	8642160	0.0231	9.88E-09
**rs473902**	9	PTCH1	T	G	98256235	0.049	4.35E-12
**rs4868125**	5	FBXW11	C	G	171281875	0.0215	2.93E-09
**rs4963975**	12	ITPR2-SSPN	A	G	26443030	0.0254	3.37E-09
**rs6012558**	20	ARFGEF2	A	G	47531286	0.0197	1.91E-08
**rs606452**	11	SERPINH1	A	C	75276178	0.0287	1.10E-08
**rs6088721**	20	EDEM2	A	C	33706011	0.0212	7.32E-09
**rs6107848**	20	BMP2	A	G	6591116	0.0238	1.49E-10
**rs615672**	6	HLA-DRB1	G	C	32574171	0.0234	3.30E-08
**rs6440003**	3	ZBTB38	A	G	141094209	0.0269	5.10E-15
**rs6470765**	8	GSDMC	A	C	130736697	0.0259	4.80E-08
**rs6549455**	3	RYBP	G	A	72457221	0.0258	9.41E-09
**rs6556301**	5	FGFR4	T	G	176527577	0.0314	3.50E-14
**rs6715793**	2	LTBP1	T	C	33379263	0.0193	1.44E-08
**rs6743226**	2	HDLBP	C	T	242236972	0.0224	1.20E-10

**rs6772896**	3	ANAPC13	T	C	134203347	0.026	1.20E-12
**rs6830062**	4	LCORL	T	C	18017730	0.0313	1.10E-10
**rs6892330**	5	MEF2C	T	C	88746405	0.0217	3.48E-08
**rs710841**	4	PRKG2	T	C	82149831	0.0262	1.19E-09
**rs7166081**	15	FLJ11506	A	G	67492301	0.0266	1.59E-10
**rs741677**	17	ACAN	A	G	463843	0.0243	1.87E-08
**rs7513580**	1	SPAG17	G	A	118849909	0.0359	7.60E-20
**rs757608**	17	ATAD5,ITPR2-SSPN	A	G	59497277	0.026	6.17E-11
**rs7621331**	3	PPP2R3A	A	G	135761927	0.0207	9.41E-09
**rs7697556**	4	ADAMTS3	T	C	73515313	0.0205	5.20E-09
**rs7759938**	6	HACE1	C	T	105378954	0.0307	1.20E-16
**rs7801581**	7	HOXA11	T	C	27223771	0.0271	8.01E-11
**rs780159**	10	LOC283050	G	A	80907147	0.0223	5.84E-09
**rs7854560**	9	PTCH1	T	C	98382950	0.025	4.00E-11
**rs7970350**	12	HMGA2	T	C	66360164	0.0188	3.76E-08
**rs798502**	7	GNA12	A	C	2789880	0.0243	2.95E-11
**rs8028537**	15	ACAN	G	A	89345947	0.0203	4.59E-08
**rs8055190**	16	LRRC36	C	T	67391618	0.0645	4.84E-13
**rs806794**	6	HIST1H2BF	A	G	26200677	0.0276	3.20E-13
**rs822531**	7	EZH2	T	C	148629759	0.0244	3.71E-08
**rs849140**	7	JAZF1	T	C	28183702	0.0288	4.74E-17
**rs893817**	15	LOXL1	G	A	74229065	0.0228	9.55E-10
**rs9409082**	9	TMEM38B	C	T	108901049	0.0268	1.50E-08
**rs9435732**	1	MFAP2	C	T	17308158	0.0318	2.50E-16

**rs984225**	1	TBX15	A	G	119504284	0.0366	3.30E-26
**rs9860730**	3	ADAMTS9	A	G	64701146	0.0219	2.07E-09
**rs9890032**	17	ATAD5	C	G	29165934	0.0224	3.22E-10
**rs991967**	1	TGFB2	C	A	218615451	0.0274	1.60E-13
**rs9977276**	21	COL6A1	T	G	47436327	0.0218	4.37E-08
**rs998584**	6	VEGFA	A	C	43757896	0.0293	6.45E-15

Reference

1. Randall JC, Winkler TW, Kutalik Z, et al. Sex-stratified genome-wide association studies including 270,000 individuals show sexual dimorphism in genetic loci for anthropometric traits. *PLoS genetics* 2013;9(6):e1003500.
